# Identifying the impediments and enablers of ecohealth for a case study on health and environmental sanitation in Hà Nam, Vietnam

**DOI:** 10.1186/2049-9957-3-36

**Published:** 2014-10-01

**Authors:** Vi Nguyen, Hung Nguyen-Viet, Phuc Pham-Duc, Craig Stephen, Scott A McEwen

**Affiliations:** Department of Population Medicine, Ontario Veterinary College, University of Guelph, 2509 Stewart Building (#45), Guelph, N1G 2W1 ON Canada; Public Health Risk Sciences Division, Laboratory for Foodborne Zoonoses, Public Health Agency of Canada, 160 Research Lane, Unit 206, Guelph, ON N1G 5B2 Canada; Center for Public Health and Ecosystem Research, Hanoi School of Public Health (HSPH), 138 Giang Vo Street, Hanoi, Vietnam; Swiss Tropical and Public Health Institute (Swiss TPH), and International Livestock Research Institute (ILRI), Socinstrasse 57, CH-4002 Basel, Switzerland and Hanoi, Vietnam; Swiss Federal Institute of Aquatic Science and Technology (ESWAG), Sandec – Department of Water and Sanitation in Developing Countries, P.O. Box, CH-8600, Dübendorf, Switzerland; Department of Ecosystem and Public Health, Faculty of Veterinary Medicine, University of Calgary, TRW 2D26 3280 Hospital Drive, NW, Calgary, Alberta T2N 4Z6 Canada; Centre for Coastal Health, 900 Fifth Street, Nanaimo, British Columbia V9R 5S5 Canada

**Keywords:** Ecohealth, Evaluation, Health, Sanitation, Case study, Vietnam

## Abstract

**Background:**

To date, research has shown an increasing use of the term “ecohealth” in literature, but few researchers have explicitly described how it has been used. We investigated a project on health and environmental sanitation (the conceptual framework of which included the pillars of ecohealth) to identify the impediments and enablers of ecohealth and investigate how it can move from concept to practice.

**Methods:**

A case study approach was used. The interview questions were centred on the nature of interactions and the sharing of information between stakeholders.

**Results:**

The analysis identified nine impediments and 15 enablers of ecohealth. Three themes relating to impediments, in particular—*integration is not clear*, *don’t understand*, and *limited participation*—related more directly to the challenges in applying the ecohealth pillars of transdisciplinarity and participation. The themes relating to enablers—*awareness and understanding*, *capacity development*, and *interactions*—facilitated usage of the research results. By extracting information on the environmental, social, economic, and health aspects of environmental sanitation, we found that the issue spanned multiple scales and sectors.

**Conclusion:**

The challenge of how to integrate these aspects should be considered at the design stage and throughout the research process. We recommend that ecohealth research teams include a self-investigation of their processes in order to facilitate a comparison of moving from concept to practice, which may offer insights into how to evaluate the process.

**Electronic supplementary material:**

The online version of this article (doi:10.1186/2049-9957-3-36) contains supplementary material, which is available to authorized users.

## Multilingual abstracts

Please see Additional file 1 for translations of the abstract into the six official working languages of the United Nations.

## Background

“Ecohealth can be defined as systemic, participatory approaches to understanding and promoting health and wellbeing in the context of social and ecological interactions” [1]. It has been built upon the approach of improving human health through integrated management of ecosystems and the understanding that health is integral to systems at different biological scales, from the individual to the biosphere [2, 3]. There is currently no consensus for an overarching paradigm or a particular set of techniques for ecohealth practice [1, 4–6]. Forget and Lebel’s [2] discussion on the history and evolution of the paradigm encompassed and elaborated on the descriptions presented above. Ecohealth is useful to address complex problems that span multiple disciplines and sectors, like many other integrated approaches, such as the Population Health Approach, the Global Health Research Initiative, the Millennium Ecosystem Assessment, and the One Health Initiative [7–10]. Recently, there has been an increasing use of the term ‘ecohealth’ in literature, yet many researchers who have used this approach have not explicitly described how they applied it [5]. A scoping review on ecohealth found that only two primary research papers explained their processes, making it difficult to review the utility of ecohealth in practice from the existing body of literature [11, 12].

Monitoring and evaluating the process of ecohealth research and its outcomes are important components of ecohealth [13]. There has been, however, relatively little published research on the evaluation of ecohealth projects, including in-progress evaluation, to determine their consistency with ecohealth concepts [14]. While Boischio and colleagues discussed the challenges and opportunities of ecosystem approaches in the prevention and control of dengue and Chagas disease, the discussion concerned their experience with the Canadian International Development Research Centre’s (IDRC) Ecohealth Program Initiative rather than being a project evaluation *per se* [15]. The IDRC has emphasized outcome mapping for ecohealth evaluation, however, this mapping is difficult to apply to projects in-progress when there is usually inadequate time to achieve project outcomes [13, 16]. Thus, a case study involving a mid-term examination of the processes used in an integrated approach may provide useful insights for understanding ecohealth’s concepts and practices. The case study approach is a well-recognized methodology in qualitative research and is useful for in-depth investigation [17].

The specific challenges and opportunities for implementing ecohealth in practice will be affected by contextual factors such as culture, national policies, infrastructure, and the nature of the problem(s) being examined. However, the implementation issues encountered when working across disciplines, using participatory approaches, ensuring equity in the process, and building capacity for the sustainability of interventions may apply more generally across ecohealth projects. A recent scoping review of the peer-reviewed literature on ecohealth revealed that the practical aspects of applying ecohealth concepts have received relatively little attention [5]. While the present investigation focused specifically on ecohealth, other integrated approaches (that are not limited to “one health”) have similar aims and also address health challenges that lie at the human, animal, and environment interface. Thus, these approaches can also benefit from the findings in this paper [18]. Zinsstag *et al*. [18] have discussed these issues through the history of integrative thinking in human and animal health, the evolution of “one medicine” towards “one health”, and the emergence of ecohealth over the past few decades in response to broader thinking in global health.

Through the Swiss National Centre for Competence in Research North–South Program (NCCR North–South), a conceptual framework for environmental sanitation assessment to improve human health and environmental sustainability was developed and tested in different settings in Southeast Asia and West Africa [19]. The project in Vietnam aimed to assess the risk of the reuse of human waste and wastewater for agriculture, environmental sanitation, and human health [19–24]. The conceptual framework for that project incorporated the following pillars of ecohealth: sustainability, participation, equity, and transdisciplinarity, as defined by the Community of Practice in Ecosystem Approaches to Health – Canada (CoPEH-Can) [25]. We aimed to identify the impediments and enablers of ecohealth in practice for a project on health and environmental sanitation and assess how well the research process fits with the concepts of ecohealth. This was accomplished by examining the nature of the interactions among stakeholders, investigating how knowledge was shared, and identifying which themes were consistent with ecohealth themes in literature and which were unique to this case.

## Methods

### Study approach

This research followed a case study structure which included: case and boundary identification, finding and assessing sources of information for data collection, and context description [17]. Our approach examined the nature of interactions among stakeholders and how information was shared through the research process. A stakeholder was defined as a person or a group of people that was affected by the issue of environmental sanitation in the project site and/or involved in the research process. Involvement was defined as participation in problem definition, establishing partnerships/collaborations, research planning, execution, analysis, or results sharing. For our purposes, the researchers were also considered to be stakeholders.

### Study design

#### Identification of the system being studied

The system being studied was confined to the research project of the NCCR North–South research team in Vietnam and the stakeholders involved. All of our case study data were collected in Vietnam by the first author. Initially, sources of information included some project documents in English and meetings with the NCCR North–South research team.

#### Selection and recruitment of participants

We selected participants by identifying the categories and identities of stakeholders through an interview with the NCCR North–South project lead. All of the four graduate student investigators identified by the project lead described the general roles of the project participants when we interviewed them. We chose the Head of the Health Station and a few health station workers and village health workers from both communes as participants because they provided population health information and have previously conducted interviews with commune residents (see Table 1). Project participants were selected from a list of all community members; they were the project’s intended beneficiaries. Female participants were purposively selected, as they were primarily responsible for family health, sanitation, and agricultural work in their villages. To capture a diversity of perspectives, they were selected from different villages by convenience sampling, depending on the availability of participants.

**Table 1:** **Case study data collection methods, languages of delivery, and purposes of questions, by stakeholder group**

	***Stakeholder group***			
***Category***	***Project lead (n = 1)***	***Graduate student researchers (n = 4)***	***Head of health station & health station workers (n = 6)***	***Village health workers & community members (n = 22)***
***Data collection method***	Key informant interviews	Key informant interviews	Key informant interviews	Focus groups
***Language***	English	All Vietnamese except Part 1 with PhD student	Vietnamese	Vietnamese
***Purpose of questions***	Stakeholder role (1*, 3**)	Stakeholder role (1*, 3**)	Respondent information (2*, 0**) – 3 for health station workers	Involvement in this research (1*, 4 **)
	Understanding the research problem (1*, 5**)	Interaction between the research team (1*, 6**)	Participation in the research (11*, 0**)	Thoughts on the research topic (1*, 14**)
	Establishing collaborations (2*, 8**)	Research objectives (2*, 4**)	Results sharing (4*, 0**)	Researchers’ approaches (1*, 8**)
	Research planning (2*, 0**)	Sharing of information (3*, 5**)	Using research results (6*, 0**)	Issues important to the community (1*, 5**)
	Conducting research (2*, 1**)	Understanding the research problem (2*, 4**)		Learning from participation (1*, 4**)
	Analyzing/interpreting results (1*, 0**)	Successes & challenges (2*, 0**)		
	Results sharing (4*, 0**)	Contribution to the community members (1*, 1**)		
	Beneficiaries of the research (3*, 0**)	Beneficiaries of the research (3*, 0**)		
	Research objectives (1*, 0**)	Research approach (9*, 0**)		
	Research approach (15*, 6**)			

*number of questions.

**number of probes for each question.

#### Data collection

The case study data were collected between January and May 2010. Table 1 lists the data collection methods, languages, and purposes of questions by stakeholder group. The entire list of interview questions with each stakeholder group was too lengthy to report here, but it is available upon request to the corresponding author. Open-ended questions solicited information on the nature of interactions among project stakeholders and how knowledge was shared. Eight participants were invited to each focus group. Eight and six participants participated in the first and second focus groups in the Nhât Tân Commune, respectively. Five and three participants participated in the first and second focus groups in the Hoang Tay Commune, respectively. All interviews and focus groups were designed to last between 1 to 1.5 hours. We conducted a total of four focus groups.

All questions were drafted in English, and then translated into Vietnamese prior to the interviews. Most interviews were conducted in Vietnamese with the assistance of a translator, while a few were conducted in English with those proficient enough in the language. Interviews were digitally recorded and responses were translated and transcribed directly into English by the translator, then checked by the primary author during analysis. Data collection commenced after approval from the University of Guelph Research Ethics Board (REB# 10JA017) and the Hanoi School of Public Health Ethical Review Board (Decision No. 010-005/DD-YTCC) was obtained.

#### Translation, transcription, and analysis

All responses were analyzed using a modification of the analysis method framework; the first step was adapted to provide guidance on coding themes and writing memos (see Table 2) [26]. After each interview, initial themes were identified by listening to the interview recordings directly after rather than waiting for the translation and transcription. The remaining steps of the analysis method framework were implemented for all transcripts following the data collection, and translation and transcription. This was managed using qualitative data analysis software, ATLAS.ti 6.1 (ATLAS.ti GmbH, Berlin, Germany).

**Table 2:** **Steps in the analysis method framework used for the analysis of interview and focus group responses**

***Step***	***Explanation***
***1***	Identifying initial themes by reading the document, writing memos about the data, and creating a coding list with definitions.
***2***	Labeling or tagging data by theme by applying the coding list to other documents and iteratively making revisions to the coding list for new themes that emerge.
***3***	Sorting data by theme, each in a separate matrix that allows the reader to clearly see the data and the document from which it came.
***4***	Summarizing and synthesizing data in another similar matrix that only captures the content and context.
***5***	Identifying elements and dimensions, refining categories, classifying data in another matrix by reading the matrices from the previous steps and labeling the data to suggest what it represents.
***6***	Detecting patterns by searching within and then across documents for linkages and repetition.
***7***	Developing explanations by giving reasons that relate to the patterns found in the previous step.

## Results

### Description of the case and its context

**Figure 1:**
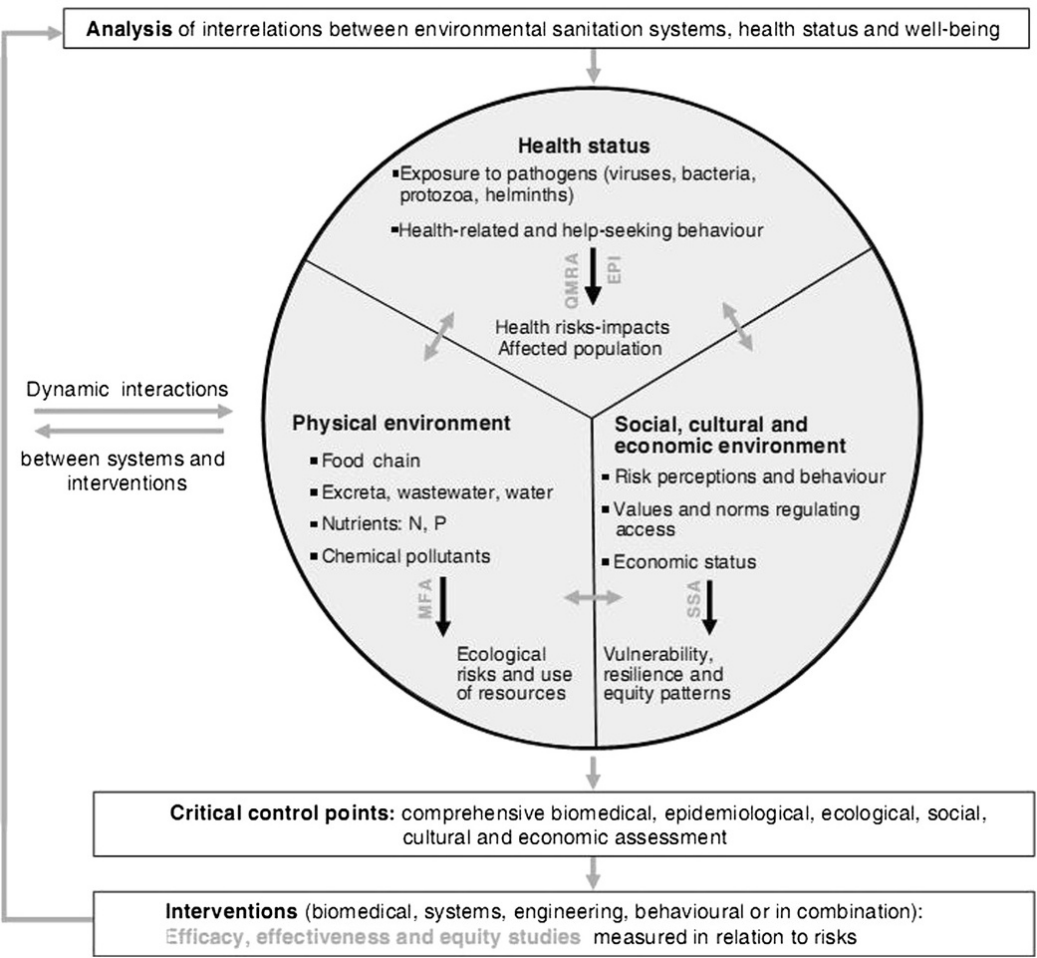
**Conceptual framework of the combination of health and an environmental risk assessment for health and environmental sanitation planning.** This was the framework of the project that we investigated. EPI: Epidemiology, QMRA: Quantitative Microbial Risk Assessment, MFA: Material Flow Analysis, SSA: Social Science Analysis.

The NCCR North–South was one of 20 programs initiated in 2001 by the Swiss National Science Foundation for sustainable development research [27]. The purpose of this 12-year program was to build research capacity in partnerships between northern and southern institutions in nine regions of Asia, Africa, Latin America, and Switzerland, while also establishing a formal institutional network in these countries. This case study was limited to Phase 2 of the research, in particular, health and environmental sanitation. The conceptual framework, developed by the NCCR North–South researchers (see Figure 1), was tested in Southeast Asia and West Africa [19]. The subject of our case study was the research process in Vietnam (part of NCCR North–South project), which assessed the risk that the reuse of human excreta and wastewater in agriculture and aquaculture poses to the environment, health, and socio-economics (hereafter referred to as “the problem”).

**Figure 2:**
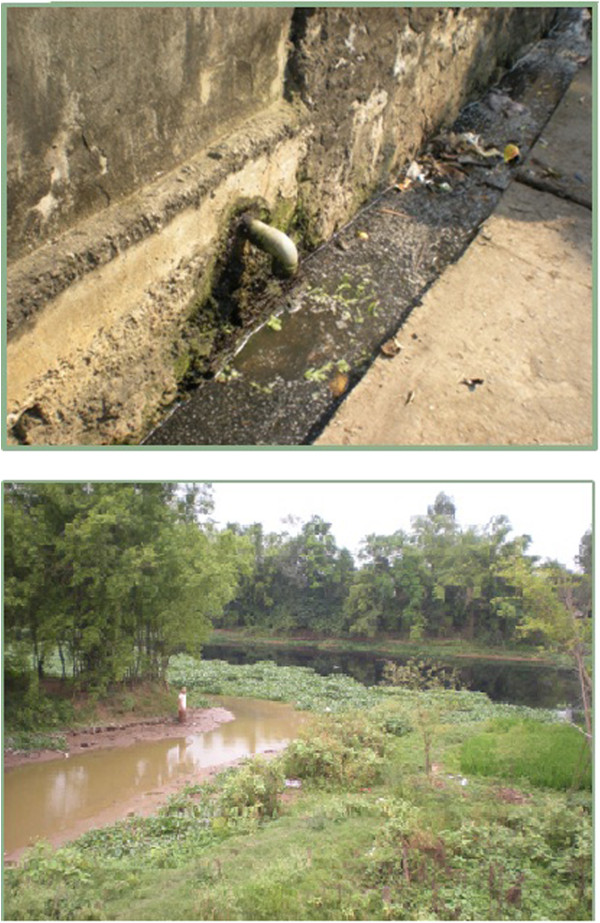
**Open drainage system (top) and Nhue River containing untreated wastewater flowing from Hanoi (bottom) in Hoang Tay Commune, Kim Bang District, Hà Nam Province, North Vietnam.** Photo: Vi Nguyen, 2010.

The project was conducted in a peri-urban area, approximately 60 km south of Hanoi, in the Nhât Tân and Hoang Tay Communes, Kim Bang District, Hà Nam Province, Vietnam. Both are typical northern Vietnamese communes, with poor services for sanitation, wastewater drainage, and solid waste management [23] (see Figure 2). Household effluent is discharged untreated and flows through dykes that end up in the Nhue River, which flows through the commune. This river, being the only agricultural irrigation source for the communes, also receives untreated effluent from Hanoi [28]. At the time of the study, there was no place for garbage disposal and as a result, rubbish often ended up on the side of the commune roads, where it was often burned. The major land uses are residential, aquaculture, and agriculture (rice cultivation and vegetables); the latter being the main source of livelihood (see Figure 2).

**Figure 3:**
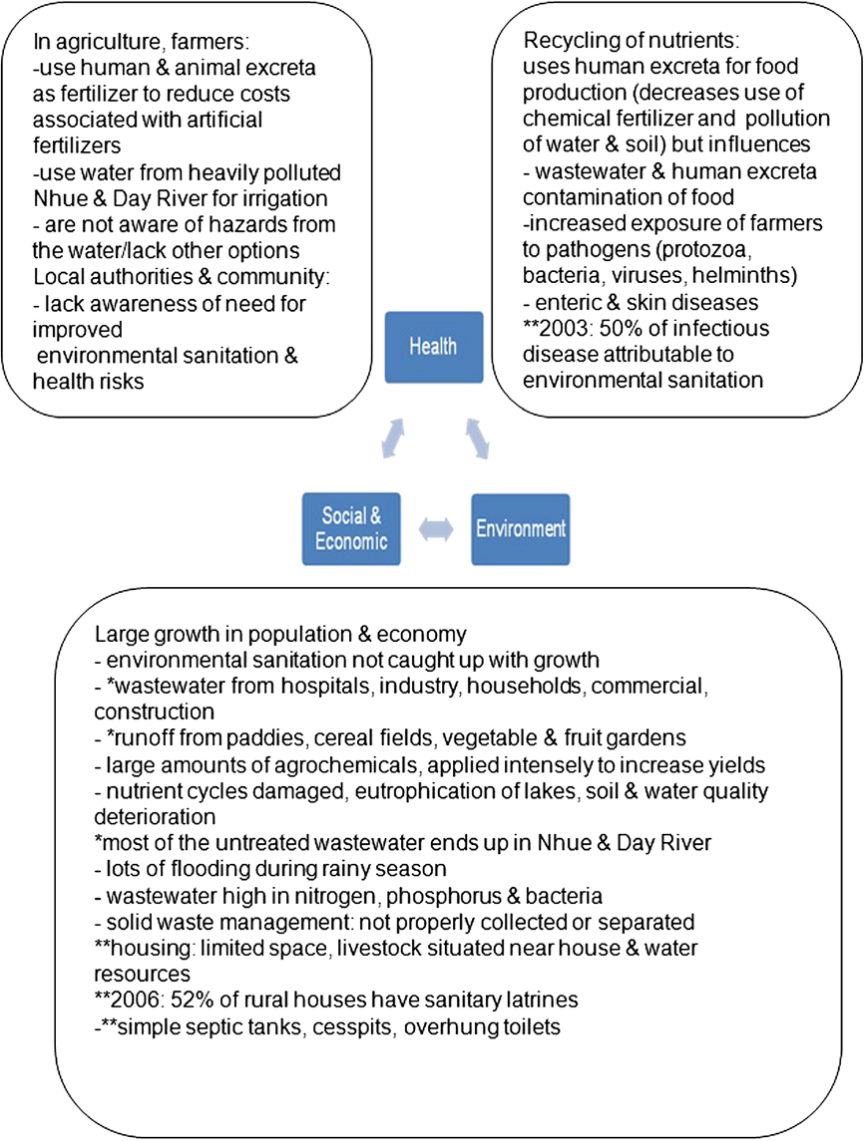
Environmental, social, economic, and health aspects of the problem from a research perspective (*from Hanoi, **in rural areas).

Figure 3 shows a broad overview of the environmental, social, economic, and health aspects of the problem, the details of which were extracted from project documents. The project stakeholders included institutions (Hà Nam Centre for Preventive Medicine, National Institute of Hygiene and Epidemiology, and the Hanoi School of Public Health), local authorities (Communal Head of the Health Station, health station workers, Communal People’s Committee, District Level Health Services, Women’s Union, and village health workers), and the NCCR North–South research team and their research participants (community members from both communes who responded to household surveys). The project involved four graduate students working on sub-projects in the same study sites. The general study details of each sub-project are shown in Table 3.

**Table 3:** **Description of the major elements of sub-projects within the health, social, and environmental research components**

	***NCCR research project component***			
***Category***	***Health***		***Social***	***Environmental***
***Degree (number of students), discipline***	PhD (1), Epidemiology	MPH (1)	MPH (1)	MSc (1), Environmental Engineering & Management
***Title***	Health risks of wastewater & excreta reuse in agriculture & aquaculture in northern Vietnam	QMRA^1^ of exposure to wastewater & excreta in agriculture in Hà Nam, Vietnam	Assessment of human behaviors of reusing wastewater & excreta in agriculture based on PMT^2^ Framework	Assessing nutrient flows by MFA^3^ in Hà Nam, Vietnam
***Objective(s)***	Determine prevalence of infections of helminths, *E. histolytica, C. parvum, G. lamblia*, & Cyclospora, incidence & risk factors of diarrheal disease	Assess exposure to wastewater & excreta in agriculture & determine the risk of infection by *C. parvum*, *G. lamblia*	Examine perception & behavior related to the use of wastewater & excreta (health risk, coping appraisal, intention to act) based on PMT, develop a questionnaire to assess this, validate the questionnaire	Quantify nutrient (N^4^ & P^5^) flows in an agricultural & environmental sanitation system, develop scenarios to reduce the N or P discharge into the environment at all critical control points
***Data collection dates***	June – October 2008, April – June 2009, August – July 2010	October 2008 – October 2009	October 2008 – October 2009	August 2008 –January 2009
***Methodologies***	Epidemiology Microbiology, Parasitology	QMRA Microbiology, Parasitology	PMT	MFA
***Data sources & collection methods***	Household surveys, human feces sampling	Wastewater sampling	Qualitative: in-depth interview, focus group discussions with farmers, field observation, quantitative surveys	Annual reports, primary research studies, working group papers, statistical records, maps, field observation, key informant/expert interviews, household surveys

^1^QMRA: Quantitative Microbial Risk Assessment; ^2^PMT: Protection Motivation Theory; ^3^MFA: Material Flow Analysis; ^4^ N: nitrogen; ^5^P: phosphorous.

### Interviews and focus groups

All of the interview themes were identified through questions about the nature of interactions and the sharing of information among stakeholders. The analysis identified nine impediments and 15 enablers of ecohealth, as shown in Table 4 and below. The themes presented in-text are not presented in the table to avoid repetition of data.

**Table 4:** **Themes categorized as enablers and impediments of ecohealth for this case study**

Category	Theme	Explanation	Selected quotations
Impediments	Lack of acceptance	People did not want to change their conventional ways of doing research	“For this school, if you look at the topic of Master’s thesis, almost all topics were done in a classical way: epidemiological survey, cross-sectional study…and what they [students] don’t want is to design a study, going to the field, taking samples like [our MSc student] to do analysis. Because [the students] are already staff in different institution so they have a database… to analyse”.
	Not comfortable talking to highly educated researchers	Differing education levels and professional backgrounds impeded communication among some stakeholders	“They [the researchers] are nice and enthusiastic but just our ability is limited. When we [Village Health Workers] meet them [we don’t feel very comfortable] because we are not highly educated, we can’t keep up with them”.
	Terminology	Lack terminology in their native language which made it hard to express ecohealth concept for others to understand	“Actually it [the Vietnamese language] doesn’t have it [the ecohealth concept] now. I, myself, can’t find any Vietnamese word for researchers to understand it clearly. Maybe if someone can combine all the ideas of those people [perspectives of ecohealth], the definition of ecohealth can be clearer”.
	Past history of extractive research	Community members expressed frustration with years of research and seeing no changes.	“The people hope that after the research is done, [researchers] will soon have solutions so that they know the situation [in our commune]. If you just come and ask many times without results, they will say ’they come here and ask many times, take the water samples but we haven’t seen any results’”.
	Lack of interaction	Difficult to maintain a relationship with stakeholders with whom they didn’t have a lot of direct interaction with	“We go regularly to meet them to update about the work… the outputs of the research…I’m talking about the health worker level because in the end you can’t have a lot of relationship with the participants from the community”.
	Differing priorities	Research that was relevant for what researcher’s deemed important did not match the nature of the problem	“For the project objective, we had to make sure it was an environmental health problem. The community’s main health problems were skin problems and diarrhoea. Microbiologists are more concerned about the chemicals -heavy metals in wastewater but our background is in the health, about the diarrheal diseases and parasitic infections. Our study objective and the main problem in the study site did not match”.
Enablers	Consensus	Agreement among groups	“Need to find compromise between you [researcher], the community, and policy-makers [to plan interventions]. But when you implement, I think we need the strong willingness of the Communal People’s Committee, Health Station, other mass organizations, and the community”.
	Equity	Accounted for differences among different groups (gender, stakeholder level, social status, etc.)	“It’s mainly the Women’s Union. If they have their meeting, I would like to have a meeting in this commune about environmental sanitation. Because they [women] are in charge of housework and going to the field. I would like to have a meeting with them because they mainly clean the road. The men don’t do it. The custom is like that”.
	Evidence	The research provided evidence that the community could use	“The people knew before that there was pollution, but now through the researchers, the main influences have been discovered. Why they are infected with helminths? Or where does the diarrhoea come from? They can be aware of that now. It was vague before”.
	Free to express concerns	Health Station Workers and community members were free to ask researchers questions if they didn’t understand the survey questions	“When they [the researchers] come, they often ask if we have any concerns [regarding research]. If yes, we will discuss with them so that it’s easier to do”.
	Funding	Financial contributions from collaborators	“We need financial support to clean and rebuild the facilities so that the environment can be improved. Without funding, the drains would never be clean”.
	A channel for concerns	Through the Health Station, the community could voice opinions to the Communal People’s Committee	“We will give our opinions to the Head of Health Station in a monthly meeting. The Health Station will collect all the opinions and submit them to the upper levels”.
	Networks	Must be well-known among those working in the area; offers access to other opportunities	“I would go to approach them [policy-makers] once I have more evidence and in particular, a bigger network…people working in the Ministry [of Health]… Environment, in the University, in the Institute. We can have some kinds of recognition when we can talk with them”.
	Pluralism	Multiple methods and perspectives, included multiple stakeholders at different levels	“With one person, the problem can’t be seen comprehensively but a group of researchers with the same idea about improving environment for health, there will be many researchers joining and thus, many ideas contributed from many sides. About research with community’s participation, if we have the participation of the community, the information will be more reliable and timely”.
	Research in partnership	Decisions on research made together among partners involved in the research	“We discuss together, identify the problem together and we will do research together with the resources we already have. We are also willing to discuss with people to find other funds, other support to support our common interest”.
	Sharing process	The responsibility for interventions, the data, and results should be shared by stakeholders; each person has a part	“Because when all unions and department co-operate, they can advocate widely to people, the people can follow, and keep good sanitation. It can’t work if just one does it. They can’t go to each person”.
	Commitment to ongoing testing and monitoring	The desire for project commitment to addressing sanitation beyond data collection and research outputs	“I also want the people from the environment section to come here and take the [water] sample for testing so that we can know. Or when you do research, you know the information and you will share information with us so that we can learn from experience”.
	Sharing knowledge gained through research	Village Health Workers shared what they have learned through the research with others in their community	“By talking, for example, with the women here (Village Health Workers) or the neighbours talk with each other or when we have a [Women’s Union] meeting”.

Three impediment themes in particular—*integration is not clear*, *don’t understand*, and *limited participation*—related more directly with the challenges in applying the ecohealth pillars of transdisciplinarity and participation. When asked about *how* and *what* was integrated in the research, a project team member explained that “*the concepts were developed with the expectation that we would integrate information for the three components… So we did it [the research]. But the integration is not clear…we need to explore further to see the link between the three components*”.

In a discussion of how the community could directly use the research results, a village health worker said that “*if the [community members] didn’t participate and just attended to listen to the results, they wouldn’t understand them. When the researchers came to present the results, they presented very briefly*”. Community members and health station workers explained that they wanted to participate in interventions to mitigate the problem as much as they could, but they felt limited by their knowledge, abilities, time, resources, and funding (for example: “*The Health Station just advocates. We have to depend on many things. We don’t have any funding. We just advocate by using loudspeakers or through the village health workers. We have also launched campaigns to collect garbage and general campaigns, but that’s all we can do. It mainly depends on the Communal People’s Committee*”).

On the other hand, the enabler themes—*awareness and understanding*, *capacity development*, and *interactions*—facilitated usage of the research results. Village health workers echoed that “*regarding the waste in the Nhue River*, *we do know about it [its effects on health], but we don’t know the percentage of the infection or pollution, whether it is too high, without the [research] results*”. A project team member said that “*NCCR North–South focuses on partnership with Vietnam’s institutes…by cooperating with foreign countries, they improve research capacity [of researchers and supporters]…learn new methods and knowledge. NCCR North–South wants them to be active in research so [they] don’t need to wait for any external support*”. Another researcher noted that there has been “*more contact with them [health station workers] every time we go [to the study site]… health station workers have much more contact and good relationships with community members. Researchers can’t cover everything*”.

Discussions with community members about solutions, community roles, and signs of improvement in health and environmental sanitation yielded input that spanned not only the health sector, but also the environmental, social, and economic aspects of the issue (see Table 5). We felt that this discussion was necessary in order to get community input on what was necessary to enable the next steps since ecohealth is so action oriented [5].

**Table 5:** **Community members’ input on the solutions, roles, and signs of improvement for health and environmental sanitation**

Community-identified ideal solutions or community roles in environmental sanitation	Community-identified signs of improvements in health and environmental sanitation
Use a biogas oven (converts waste into fuel)	Cleaner roads (no more garbage thrown randomly)
Burn garbage	Everyone gathers household garbage for a garbage collector; identified the need for regulations
Treat excreta to get rid of smell or compost it properly	Economic status is better
Lead by example by making changes and other people will follow if they see changes working	Improved health means we can do anything
Need funding	Reduction in diseases and conditions they perceived to result from poor sanitation (diarrheal diseases, skin diseases, cancer)
Need awareness & understanding	No smell (from garbage, animal carcasses thrown into the river, and the wastewater itself)
Need a clean water system and wastewater treatment system	No wastewater visible (for human exposure)

We assessed the project’s consistency with ecohealth concepts identified in the scoping review [5] (see Table 6). The comparison with project details and interview themes revealed that the main challenges were related to limited participation and how to integrate research components. The strengths of the project were: the timeframe, which showed a long-term commitment (from 2008 and continuing through to 2013 and beyond) to health and environmental sanitation in the community, and that multiple disciplines and research questions examining the different aspects of the issue attempted to address its complexity.

**Table 6:** **Assessment of the case study’s consistency with ecohealth components identified in the scoping review of ecohealth**

Ecohealth component	Component explanation	Corresponding project elements	Source of information
Participation	- from the beginning, stakeholders (including affected population) collaborate on various research stages using local knowledge and addressing some of their priorities; also refers to participatory action research	- participation from member of local institutions and community members consisted of providing information for the researchers’ project and helping them collect data	- interview theme: “limited participation” (Table 4)
System	- understanding the whole and its parts (issues, interactions, key actors, components, and interrelationships); includes systems science	- not be evaluated at the time of this study^1^	N/A
Multidisciplinary	- more than two disciplines working together in their traditional roles	- More than one discipline was involved (epidemiology/public health, environmental engineering) but all were allied health professions	- project documents (Table 3)
Action-oriented	- results in something done to solve or mitigate the research problem under study	- no interventions or changes were planned at the time of this study but they intended to address this in the next phase of research	- interview with project lead (interview transcript, not shown here)
Complexity	- made up of many interrelated parts; where ecohealth is best applicable	- the project was designed to address several dimensions of the sanitation problem and made efforts to share results and perspectives across disciplines and stakeholders	- project documents (Table 3 and Figure 3)
Long-term	- ecohealth requires a time-commitment; improvements/outcomes might only be seen in the future; difficult to contain within a single project	- data collection started in 2008; next phase of research was expected to last until 2013	- project documents (Table 3)
		- project involved multiple components	
Indicators	- measures used for study outcomes and monitoring should be developed by involved stakeholders and may be different according to each group	- community-identified indicators had not been discussed with the researchers or addressed at the time of this project	- “community identified signs of improvement” (Table 5)
Adaptive management	- an iterative learning process with stakeholder participation involving monitoring, evaluating, and adjusting the plan based on the information generated in the process	- could not tell at the time of this study^1^	N/A
Transdisciplinarity	- collaboration between researchers and practitioners from complimentary disciplines/sectors and/or other stakeholders on a problem; uses multiple methods/tools that facilitate the generation of new frameworks, concepts, methods, institutions, etc. from the knowledge sharing and/or interaction	- integration of research components was not clear; integration of results was anticipated, but how this will happen was not clear	- interview theme: “integration is not clear” (Table 4)
Equity	- addresses differences between groups affected by research problem; gender (roles, responsibilities), power (decision making, access to resources), and trade-offs (who benefits)	- statistical analysis of data had been stratified by gender	- interview with PhD student on health research component (interview transcript, not shown here)
Sustainability	- meeting the needs of current generations without compromising the needs of future generations; the outcome or goal of ecohealth, also refers to sustainability of the environment and/or of interventions/projects	- could not tell at the time of this study^1^	N/A
Socio-ecological	- understanding the human and environmental components of a problem and their interaction	- health component quantifies human health risks and exposure	- project document (Table 3)
		- social component examines perceptions & behaviours	- interview theme: “integration is not clear” (Table 4)
		- environmental component quantifies nutrient flows in agricultural & sanitation system	
		- the interaction between components not addressed yet, as integration is not clear	
SOHOs (self-organizing, holarchic open system)	- characterized by holarchy (interactions between nested hierarchies), feedback loops (consequences for another part of the system – positive or negative), self-organization (combination of feedback, boundaries, and openness)	- could not tell at this point in the project^1^	N/A
Negotiate	- a process in which the decisions on objectives, methods, and indicators are made with stakeholders	- the research was conducted according to researchers’ priorities, mainly driven by a conceptual framework developed *a priori*	- interview theme: “priorities” (Table 4)
			- project document (Figure 1)

## Discussion

Overall, examining the factors that helped or hindered the research team to reach an ecohealth process during the first three years of the project allowed us to identify some enablers and impediments that can help turn the theoretical components of ecohealth into practice. The project we examined was still in-progress during our study period, therefore, our findings do not reflect the entire project. While the case study project faced several challenges in implementing a number of ecohealth concepts, its conceptual framework corresponded quite strongly to ecohealth. This was evident in the design and preliminary documents, where concepts of integration, multi-stakeholder participation, and an understanding of the system were stressed. The main challenges were related to fully realizing a transdisciplinary and participatory approach, and sustaining research efforts. If our assessment was treated like a checklist, then the project could be consistent with most of the pillars of ecohealth. However, when taking in an assessment of ‘if’ or ‘how’ these components were implemented, the project faced challenges in fully realizing these themes in practice.

In terms of enablers of the research approach, an important aspect that we didn’t consider initially was the baseline to which we would compare this project. If we consider the pillars of ecohealth as defined by the IDRC as the gold standard but we don’t clearly know what that gold standard looks like in practice (in terms of methods and tools), then the best we can do is compare the research approach to a baseline of how research linking environment and health had previously been done in similar contexts, and then document the progress. That being said, the NCCR North–South research project did make efforts to address the sanitation issue from the perspective of other disciplines, to present research results back to the local institutions and community participants, and showed continued commitment to the issue and the particular study sites (see Table 6, enabler themes presented in our Results, and the Ecohealth Field Building Initiative discussed below). It is also important to consider this progress in the context of the history of ecohealth in the region. Ecohealth is relatively new in Southeast Asia compared to Latin America, for example, in terms of the development of a community of practice and research capacity [29, 30].

The case study showed that the integration aspect of transdisciplinarity was difficult to achieve. The NCCR North–South researchers collected data from different sectors, but they faced challenges integrating these data. This is a common problem for ecohealth research [6]. By extracting information on the environmental, social, economic, and health aspects of environmental sanitation, we found that the issue was not confined to a particular scale or sector, but was interconnected and spanned multiples scales (local, regional, and national) and sectors (health, social, economic, and environment). This complexity is typical of many public health problems when their multidimensional natures are adequately taken into account [12]. The need to accommodate multiple scales and sectors is a common feature of complex public health problems. For example, Marko *et al*. developed and applied a framework for analyzing the impacts of urban transportation in Edmonton, Canada and illustrated the economic, socio-cultural, infrastructural, and political factors that affected or were affected by transportation [31]. Murray and Sanchez-Choy conducted research on improving health in rural Amazonian communities, and found that in order to make connections between ecosystem variables, use of resources, and health, it was necessary to analyze the issues at the ecosystem, community, and household levels [32]. While it is acknowledged that complex problems span multiple scales and/or sectors, research should include the collection of data from the scales and sectors influencing the issue being studied. However, as illustrated by this study, there remain significant challenges in developing acceptable and effective means to integrate across disciplines and scales. Recently, Wilcox *et al*. [33] have summarized and described identifiable components of an integrative research project in the context of conservation medicine, which included: making integration part of the project; a clear research question and project goal; inclusion of disciplines; an integrative theory, model, or approach; an operational efficacy; an institutional environment conducive to collective learning; and a project plan (see Table 2.2 in their paper).

The response “don’t understand” reflects that affected stakeholders might have not been equally involved. This lack of understanding could have affected their capacity to learn from and use the research results. This response also highlights that the use of disciplinary methods (e.g. epidemiological surveys) may have limited the participation (another theme) of many stakeholders to help the researchers collect data and provide research inputs. This may have long-term consequences of “research fatigue” if the desired outcomes and expectations are not met. Tools and group processes to facilitate integration, including participatory methods that are not specific to a particular discipline, sector, or education level, may help to overcome this impediment in practice. These may include creating rich picture maps [11], or issue and influence diagrams [12] to develop a shared understanding of the issue being studied. Similar to Mertens *et al*., ecohealth practitioners should strive for collaborative (jointly determining priorities) and collegial participation (knowledge exchange yielding new understandings and locally-controlled action plans) by negotiating research priorities during planning phases and sharing research progress more regularly so that community members can participate in robust results dissemination planning in their own communities [34, 35].

The themes “awareness and understanding”, “capacity development” at the institutional level, and increased “interactions” among stakeholders highlight some of the challenges of achieving sustainability of the research efforts. These features of research impact are often not captured as research outcomes, as publications generally focus on the technical aspects of the research. Outcome mapping, an evaluation tool promoted and used by the IDRC for programs, projects, and organizations, could be used to capture these other features of ecohealth research [36, 37]. At the time of writing this paper, the research team in Vietnam was undertaking the Ecohealth Field Building Leadership Initiative (FBLI) in Southeast Asia, which was focused on research, training, policy, and networking (personal communication with HNV, principal investigator of this initiative). Their research focus was on human health issues associated with agricultural intensification, with research activities in Vietnam focused on the same study site as the NCCR North–South. Their intention was to build on past efforts and lessons learned, which showed a continued commitment to addressing the issues (linking health and the environment) affecting the community. They have implemented a field intervention examining how the combination of human and animal excreta composting influences helminth egg die-off in excreta, while maintaining its nutrient value [38]. The intervention aimed to improve the current storage practices of human excreta and to identify the best option for the safe use of excreta in agriculture. The preliminary results have been reported by Nguyen-Viet *et al*. in [38]. In addition, the NCCR North–South research was the basis from which to launch Vietnam’s One Health-Ecohealth Newsletter, as well as Vietnam’s One Health University Network (VOHUN) and FBLI.

Negotiation, as a component of ecohealth, included negotiating indicators of the successes of the research [5]. The input from community members on solutions, roles, and signs of improvement, with respect to the problem of sanitation, showed that their participation in interventions required the involvement of multiple sectors and a holistic view of health (see Table 5). This broader view of health was evident in the case study as the signs of improvement encompassed many determinants of health that lie outside of the health sector, such as economic status and the physical environment [39]. There were differences in priorities across these various determinants of health. For example, on the one hand, public health professionals have traditionally viewed improvements in health in terms of morbidity or mortality indicators (for example, reduction of diarrheal diseases). On the other hand, communities seemed more interested in cleaner roads and improved economic statuses, as identified in our case study (see Table 5). Therefore, indicators of improvements in the problem being studied need to be negotiated in ecohealth research, as our scoping review found [5].

Our study was one of few that examined how a research project could implement ecohealth components. Insights from this work could be used to inform other ecohealth projects in their planning and implementation phases. We used our synthesized interpretation of ecohealth, which was informed by a scoping review of the literature on ecohealth to assess the case study project’s consistency with ecohealth concepts [5]. This was strongly influenced by the IDRC’s position on ecohealth, as most of the published research was supported by this funder or they cited use of IDRC’s approach to ecohealth [5]. There is currently no consensus on ecohealth concepts among fields that have similar initiatives of working towards more holistic, integrated approaches (e.g. “one health” initiatives, global health research, conservation medicine, and ecosystem management), and application of these concepts is often context-specific [10, 40–42]. As a result, the understanding of what is meant by ecohealth and its implementation is varied; this particular finding was also cited by the authors of an external review of the IDRC’s Ecohealth Program [43]. An explanation of the process as it was implemented is required, as it is not intuitive, to give readers the ability to understand and evaluate a study that is classified as ecohealth. Future research should concentrate on the reporting and evaluation of processes to more rigorously guide ecohealth to develop from concept to practice.

## Conclusion

Our case study offered insights into the operational challenges that occurred when attempting to implement ecohealth. Three impediment themes in particular— *integration is not clear*, *don’t understand*, and *limited participation*—related more directly with the challenges in applying the ecohealth pillars of transdisciplinarity and participation. The enabler themes—*awareness and understanding*, *capacity development*, and *interactions*— facilitated usage of the research results. As there are many integrated approaches with similar aims to ecohealth, these challenges may apply more generally to interventions for health problems that arise at the human, animal, and environment interface. Components of ecohealth should not be treated as a checklist for inclusion. Monitoring processes and progress may also offer insights into how to evaluate ecohealth research, as it would emphasize articulation of the research approach and how implementation corresponds with concepts. Further research stemming from these lessons and insights for research design would contribute to the development of the field of ecohealth.

## Electronic supplementary material

Additional file 1: **Multilingual abstracts in the six official working languages of the United Nations.** (PDF 240 KB)

## Abbreviations

IDRC: International Development Research Centre
NCCR: North–South National Centre for Competence in Research North–South Program
CoPEH-Can: Community of Practice in Ecosystem Approaches to Health – Canada.

## References

[1] Waltner-Toews D (2009). Eco-health: a primer for veterinarians. Can Vet J.

[2] Forget G, Lebel J (2001). An ecosystem approach to health. Int J Occup Env Heal.

[3] Lebel J (2003). Health: An Ecosystem Approach.

[4] Webb J, Mergler D, Parkes MW, Saint-Charles J, Spiegel J, Waltner-Toews D, Yassi A, Woollard RF (2010). Tools for thoughtful action: the role of ecosystem approaches to health in enhancing public health. Can J Public Health.

[5] Nguyen V (2011). Understanding the Concept and Practice of Ecosystem Approaches to Health Within the Context of Public Health. MSc Thesis.

[6] Charron DF (2012). Ecohealth Research in Practice: Innovative Applications of an Ecosystem Approach to Health, Insight and Innovation in Development.

[7] CIHR: **Global health - healthy Canadians in a healthy world. Canadian institutes of health research (CIHR).** [http://www.cihr-irsc.gc.ca/e/35878.html]

[8] Corvalan C, Hales S, McMichael A (2005). Ecosystems and human wellbeing: Health synthesis.

[9] PHAC: **What is a population health approach?** [http://www.phac-aspc.gc.ca/ph-sp/approach-approche/index-eng.php]

[10] PHAC (2009). One World One Health™: From ideas to action. Report of the expert consultation. March 16–19, 2009 – Winnipeg, Manitoba, Canada.

[11] Bunch M (2003). Soft systems methodology and the ecosystem approach: a system study of the cooum river and evirons in Chennai, India. Environ Manage.

[12] Neudoerffer RC, Waltner-Toews D, Kay JJ, Joshi DD, Tamang MS (2005). A diagrammatic approach to understanding complex eco-social interactions in Kathmandu. Nepal Ecol Soc.

[13] IDRC: **Evaluation strategy (2005–2010).** [https://idl-bnc.idrc.ca/dspace/bitstream/10625/26668/1/122276.pdf]

[14] Sherwood S, Cole D, Crissman C (2007). Cultural encounters: Learning from cross-disciplinary science and development practice in ecosystem health. Dev Pract.

[15] Boischio A, Sánchez A, Orosz Z, Charron D (2009). Health and sustainable development: challenges and opportunities of ecosystem approaches in prevention and control of dengue and Chagas disease. Cad de Saúde Pública.

[16] IDRC: **Findings Brief - External Review of the Ecosystem Approaches to Health Program International Development Research Centre (IDRC).** [http://www.idrc.ca/EN/Documents/External-Review-of-the-Ecosystem-Approaches.pdf]

[17] Creswell J (2007). Qualitative Inquiry & Research Design: Choosing Among Five Approaches.

[18] Zinsstag J, E S, Waltner-Toews D, M T (2011). From “one medicine” to “one health” and systemic approaches to health and well-being. Prev Vet Med.

[19] Nguyen-Viet H, Zinsstag J, Schertenleib R, Zurbrügg C, Obrist B, Montangero A, Surkinkul N, Koné D, Morel A, Cissé G, Koottatep T, Bonfoh B, Tanner M (2009). Improving environmental sanitation, health, and well-being: a conceptual framework for integral interventions. Ecohealth.

[20] Minh HV, Nguyen-Viet H, Thanh NH, Jui-Chen Y (2013). Assessing willingness to pay for improved sanitation in rural Vietnam.

[21] Pham-Duc P, Nguyen-Viet H, Hattendorf J, Zinsstag J, Cam PD, Odermatt P (2013). Ascaris lumbricoides and Trichuris trichiura infections associated with wastewater and human excreta use in agriculture in Vietnam. Parasitol.

[22] Pham-Duc P, Nguyen-Viet H, Hattendorf J, Zinsstag J, Cam PD, Odermatt P (2011). Risk factors for *Entamoeba histolytica* infection in an agricultural community in Hanam province. Vietnam Parasit Vectors.

[23] Do-Thu N, Morel A, Nguyen-Viet H, Pham-Duc P, Nishida K, Kootattep T (2011). Assessing nutrient fluxes in a Vietnamese rural area despite limited and high uncertainty data. Resour Conserv Recy.

[24] Vu-Van T, Pham-Duc P, Nguyen NH, Tamas A, Zurbrügg C (2010). Improving farmers’ wastewater handling practice in Vietnam. Sandec News.

[25] CoPEH-Can: **Canadian Community of Practice in Ecosystem Approaches to Health (CoPEH-Can).** [http://www.copeh-canada.org/index_en.php]

[26] Spencer L, Ritchie J, O’Connor W, Ritchie J, Lewis J (2003). Analysis: practices, principles, and processes. Qualitative research practice: A guide for social science researchers.

[27] NCCR North-South (2009). Research partnerships for sustainable development in Southeast Asia: Highlights of the National Centre for Competence in Research North–South (NCCR North–South) Program in Southeast Asia, 2005–2009.

[28] MONRE, ICEM (2007). Improving Water Quality in the Day/Nhue River Basin: Capacity Building and Pollution Sources Inventory. Report No. ADB/MARD/MONRE/Project 3892-VIE.

[29] CoPEH-LAC: **What is CoPEHs-LAC? (Community of Practice in Ecosystem Approaches to Health - Latin America and the Caribbean).** [http://www.copehlac.una.ac.cr/index.php?option=com_content&view=article&id=48&Itemid=98]

[30] VSF-VWB: **Community of Practice in Ecohealth – South and Southeast Asia CoPEH- SSEA. Veterinarians Without Borders - Vétérinaires Sans Frontières (VSF-VWB).** [https://sites.google.com/site/veterinairessansfrontieres/about-copeh]

[31] Marko J, Soskolne CL, Church J, Francescutti LH, Anielski M (2004). Development and application of a framework for analysing the impacts of urban transportation. Ecohealth.

[32] Murray TP, Sanchez-Choy J (2001). Health, biodiversity, and natural resource use on the amazon frontier: an ecosystem approach. Cad de Saúde Pública.

[33] Wilcox BA, Aguirre AA, Horwitz P, Aguirre AA, Ostfeld RS, Daszak P (2012). Ecohealth: Connecting Ecology, Health and Sustainability. New Directions in Conservation Medicine.

[34] Biggs S (1989). Resource-Poor Farmer Participation in Research: A Synthesis of Experiences from Nine National Agricultural Research Systems.

[35] Mertens F, Saint-Charles J, Mergler D, Passos CJ, Lucotte M (2005). Network approach for analysing and promoting equity in participatory ecohealth research. Ecohealth.

[36] IDRC: **Outcome mapping.** [http://www.idrc.ca/en/ev-26586-201-1-DO_TOPIC.html]

[37] Anonymous: **Outcome mapping learning community.** [http://www.outcomemapping.ca/]

[38] Nguyen-Viet H, Pham-Duc P, Nguyen V, Tanner M, Vu-Van T, Van-Minh H, Zurbrüg C, Schelling E, Zinsstag J: **Chapter B5: A One Health Perspective for Integrated Human and Animal Sanitation and Nutrient Recycling.** In *One Health: The Theory and Practice of Integrated Health Approaches*. Edited by: Zinsstag J, Schelling E, Whittaker M, Tanner M, Waltner-Toews D. London: CABI; in press

[39] WHO: **The determinants of health. World Health Organization (WHO).** [http://www.who.int/hia/evidence/doh/en/index.html] PMC2626491 16462974

[40] Brown K, Mackensen J, Rosendo S (2005). Chapter 15: Integrated Responses. Ecosystems and human well-being: Policy Responses, Volume 3.

[41] Stephen C, Daibes I (2010). Defining features of the practice of global health research: an examination of 14 global health research teams. Glob Health Action.

[42] Tabor G, Aguirre AA, Ostfeld RS, Tabor CM, House C, Pearl MC (2002). Defining Conservation Medicine. Conservation medicine: Ecological health in practice.

[43] Finkelman J, MacPherson N, Silbergeld E, Zinstaag J (2008). External review of the IDRC Ecohealth Program Initiative: Final report.

