# Supplementary material for: Identifying the impediments and enablers of ecohealth for a case study on health and environmental sanitation in Hà Nam, Vietnam
Source: Infect Dis Poverty. 2014 Oct 1;3:36. doi: 10.1186/2049-9957-3-36 (PMC4194379; doi:10.1186/2049-9957-3-36)
Supplement: Supplementary file 1 — Additional file 1: Multilingual abstracts in the six official working languages of the United Nations. (PDF 240 KB) [file 40249_2014_77_MOESM1_ESM.pdf]

Translation of the abstract into the six official working languages of the United Nations

تحديد العوائق والعوامل المساعدة لـecohealth لدراسة حالة على الصحة والإصحاح البيئي في ها نام، فيتنام

نجوين، في، نجوين-نام هونج، فام دوك، فوك، ستيفن، كريج، ماكوين، سكوت أ

الملخص

خلفية: أظهرت الأبحاث التي تمت حتى الوقت الحالي زيادة استخدام مصطلح "ecohealth" في إجمالي ما نشر في الموضوع محل الدراسة، لكن عدد قليل من الباحثين وصف صراحة كيف تم استخدامه. قمنا ببحث مشروع خاص بالصحة والإصحاح البيئي (الإطار المفاهيمي الذي ضم أركان ecohealth) لتحديد العوائق والعوامل المساعدة لـecohealth ومعرفة كيف يمكن الانتقال من الجانب النظري للممارسة العملية.

الأساليب: تم استخدام منهج دراسة الحالة. تركزت أسئلة المقابلة على طبيعة التفاعل وتبادل المعلومات بين الجهات المعنية.

النتائج: حدد التحليل تسعة عوائق و15 عاملا مساعدا لـ ecohealth. ثلاث إجابات متعلقة بالعوائق، ولا سيما بالاندماج، كانت غير واضح، ولا يفهم، ومشاركة محدودة – تتعلق بشكل أكثر مباشرة بالتحديات في تطبيق ركائز ecohealth للعبرمناهجية والمشاركة. الردود المتعلقة بالعوامل المساعدة - الوعي والفهم، وتنمية القدرات، والتفاعلات - سهلت استخدام نتائج البحث. وعن طريق استخراج المعلومات عن الجوانب الاجتماعية، وجوانب الصحة البيئية والجوانب الاقتصادية للإصحاح البيئي، وجدنا أن المسألة تفرعت لعدة مقاييس وقطاعات.

الاستنتاج: ينبغي وضع التحدي المتمثل في كيفية دمج هذه الجوانب في مرحلة التصميم وطوال عملية البحث في الاعتبار. نوصي بأن أن تتحقق فرق بحث ecohealth بنفسها من عملياتها من أجل تسهيل إجراء مقارنة بين الانتقال من الجانب النظري للممارسة العملية، والتي قد توفر أفكارا حول كيفية تقييم هذه العملية.

Translated from English version into Arabic by Mahmoud Sami, through

TRANSLATORS
WITHOUT BORDERS

通过越南 Hà Nam 的一个健康环境卫生项目验证生态卫生的阻碍因素和促进因素

Nguyen, Vi, Nguyen-Viet, Hung, Pham-Duc, Phuc, Stephen, Craig, and McEwen, Scott A

摘要

引言：近日，研究显示“生态卫生”一词在文献中使用频率上升，但很少有研究者明确表述在何种情况下使用该词。本研究通过调查一个健康和环境卫生项目（该项目的概念框架包含生态卫生的核心内容）验证“生态卫生”该词的阻碍因素和促进因素。并研究如何从概念到实践践行这一概念。

方法：使用个案分析的方法，面试问题集中于讨论利益相关者之间的信息交互和共享的本质所在。

结果：该分析确定了 9 项生态卫生的阻碍因素和 15 项促进因素。3 种与障碍因素有关的反应，在特定的集合中表现为不清楚、不明白和参与有限；这些反应与运用生态卫生的核心内容（跨学科和参与）的挑战直接相关。与促进因素有关的反应，表现为认识和理解，开发能力以及对研究结果的相互交流、促进。通过提取信息环境、社会、经济、环境卫生和健康方面,我们发现该议题跨越了众多领域和行业。

结论：对于如何整合这些内容的挑战，必须在设计阶段和在整个研究过程中进行认真考虑。我们建议生态卫生研究团队设计时需包含本研究流程的自身调查,以促进从概念到实践的比较,该比较可提示如何正确评估该研究流程。

Translated from English version into Chinese by Zheng Qi, through

CHINESE CENTER FOR DISEASE CONTROL AND PREVENTION
NATIONAL INSTITUTE OF PARASITIC DISEASES

Identifier les obstacles et les facilitateurs d'éco-santé pour une étude de cas sur la santé et l'assainissement environnemental à Hà Nam, Vietnam

Nguyen, Vi, Nguyen-Viet, Hung, Pham-Duc, Phuc, Stephen, Craig, and McEwen, Scott A

Résumé

Contexte: De nos jours, la recherche a montre une augmentation de l'utilisation du terme "éco-santé" dans la littérature, mais peu de chercheurs ont décrit explicitement la façon dont il est utilisé. Nous avons étudié un projet sur la santé et l'assainissement de l'environnement (Le cadre conceptuel de ce qui inclue les piliers d'éco-santé) afin d'identifier les obstacles et les facilitateurs de l'éco-santé et étudier comment cela peut passer de concept (de la théorie) à la pratique.

Méthodes: On a utilisé une étude de cas comme approche. Les questions de l'interview étaient centrées sur la nature des interactions et le partage d'informations entre les parties prenantes.

Résultats: L'analyse a identifie 9 obstacles et 15 facilitateurs d'éco-santé. 3 réponses relatives aux obstacles, en particulier — L'intégration n'est pas Claire, pas comprise et participation limitée— Reliées plus directement aux défis d'application des piliers de transdisciplinarité d'éco-santé et de participation. Les réponses relatives aux facilitateurs — sensibilisation et compréhension, capacité de développent — une facilitation de l'usage des résultants de la recherche. En extrayant des informations sur les aspects environnementaux, sociaux, économiques et sur les aspects sanitaire de l'assainissement de l'environnement, On a trouve que le problème s'étendait sur de multiples échelles et secteurs.

Conclusion: Le défi d'intégration de ces aspects devrait être considéré lors de la phase conceptuelle et tout au long du processus de recherche. Nous recommandons que les équipes de recherche sur l'éco-santé incluent une

auto-évaluation de leurs processus afin de faciliter une comparaison entre la phase conceptuelle et la phase pratique, ce qui peut offrir des aperçus sur la façon d'évaluer le processus.

Translated from English version into French by Ricardo Henri, through

Выявление препятствий и движущих сил экологического здоровья для предметного исследования повышения состояния здоровья и окружающей среды в провинции Ханам, Вьетнам

Ви Нгуен, Хунг Нгуен-Вьет, Фук Фам-Дук, Крейг Стивен и Скотт А. Макэвен

Резюме

Краткое описание: Проведенные исследования показали рост использования термина «экологическое здоровье» в литературе, но лишь некоторые ученые открыто описали способ его использования. Мы изучили проект по повышению состояния здоровья и окружающей среды (концептуальная структура которого включала принципы экологического здоровья) в целях выявления препятствий и движущих сил экологического здоровья и изучения того, как можно перейти от концепции к практике.

Методология: Был использован подход предметного исследования. Вопросы интервью касались, главным образом, природы взаимодействий и обмена информацией между действующими лицами.

Результаты: Анализ выявил девять препятствий и 15 движущих сил экологического здоровья. Три ответа, касающихся препятствий (не понятна интеграция, не понимаю вопроса и ограниченное участие), относились непосредственно к проблемам при применении принципов экологического здоровья в рамках междисциплинарных исследований и участия. Ответы, касающиеся движущих факторов (осведомленность и понимание, развитие потенциала и взаимодействие), позволили использовать результаты исследования. Получив данные об экологических, социальных, экономических и медицинских аспектах улучшения состояния экологической среды, мы обнаружили, что проблема охватывает множество уровней и секторов.

Вывод: Проблема интеграции этих аспектов должна рассматриваться на этапе проектирования и в рамках всего исследовательского процесса. Мы рекомендуем, чтобы команды, исследующие проблему экологического здоровья, самостоятельно исследовали процессы с целью сравнения концепции и ее практической реализации, что может обеспечить лучшее понимание требований оценки процесса.

Translated from English version into Russian by Irina Zayonchkovskaya, through

Identificación de los impedimentos y facilitadores de la ecoepidemiología para un estudio de caso sobre saneamiento ambiental en Hà Nam, Vietnam

Nguyen, Vi, Nguyen-Viet, Hung, Pham-Duc, Phuc, Stephen, Craig, and McEwen, Scott A

Resumen

Antecedentes: Las investigaciones hasta la fecha muestran un aumento del uso del término "ecoepidemiología" en la literatura, sin embargo pocos investigadores han hecho una descripción explícita de su utilización. Hemos investigado un proyecto sobre salud y saneamiento ambiental (cuyo marco conceptual incluye los pilares de la ecoepidemiología), con el fin de identificar los impedimentos y facilitadores de la misma e indagar cómo podría pasar del concepto a la práctica.

Métodos: Se ha empleado un enfoque de estudio de caso. Las preguntas de la entrevista se centraron en la naturaleza de las interacciones y el intercambio de información entre las partes implicadas.

Resultados: El análisis identificó nueve impedimentos y 15 facilitadores de la ecoepidemiología. Sobre todo tres respuestas relativas a los impedimentos—no hay integración clara, no comprende y participación reducida—estaban más directamente relacionadas con los retos derivados de la aplicación de pilares de la ecoepidemiología como transdisciplinaridad y participación. Las respuestas relativas a los facilitadores—concienciación y comprensión, desarrollo de capacidades e interacciones—facilitaron el uso de los resultados de la investigación. Al extraer información de los aspectos ambientales, sociales, económicos y sanitarios del saneamiento ambiental, hallamos que la cuestión abarcaba múltiples niveles y sectores.

Conclusión: El reto sobre como integrar dichos aspectos se tomará en consideración en una fase de diseño y durante todo el proceso de investigación. Sugerimos que los equipos de investigación en ecoepidemiología incluyan una autoinvestigación de sus procedimientos con el fin de facilitar una comparación en el paso del concepto a la práctica, lo que podría ofrecer perspectivas sobre cómo evaluar el proceso.

Translated from English version into Spanish by Raquel Bentué, through
